# Fine-tuning the onset of myogenesis by homeobox proteins that interact with the *Myf5* limb enhancer

**DOI:** 10.1242/bio.014068

**Published:** 2015-11-04

**Authors:** Philippe Daubas, Nathalie Duval, Lola Bajard, Francina Langa Vives, Benoît Robert, Baljinder S. Mankoo, Margaret Buckingham

**Affiliations:** 1 CNRS URA 2578, Department of Developmental and Stem Cell Biology, Institut Pasteur, Paris 75015, France; 2 Centre d'Ingénierie génétique murine, Institut Pasteur, Paris 75015, France; 3 King's College London, Randall Division of Cell and Molecular Biophysics, New Hunt's House, Guy's Campus, London SE1 1UL, UK

**Keywords:** *Myf5* transcription, Msx1, Meox2, Mouse embryo, Limb myogenesis

## Abstract

Skeletal myogenesis in vertebrates is initiated at different sites of skeletal muscle formation during development, by activation of specific control elements of the myogenic regulatory genes. In the mouse embryo, *Myf5* is the first myogenic determination gene to be expressed and its spatiotemporal regulation requires multiple enhancer sequences, extending over 120 kb upstream of the *Mrf4-Myf5* locus. An enhancer, located at −57/−58 kb from *Myf5*, is responsible for its activation in myogenic cells derived from the hypaxial domain of the somite, that will form limb muscles. Pax3 and Six1/4 transcription factors are essential activators of this enhancer, acting on a 145-bp core element. Myogenic progenitor cells that will form the future muscle masses of the limbs express the factors necessary for *Myf5* activation when they delaminate from the hypaxial dermomyotome and migrate into the forelimb bud, however they do not activate *Myf5* and the myogenic programme until they have populated the prospective muscle masses. We show that Msx1 and Meox2 homeodomain-containing transcription factors bind *in vitro* and *in vivo* to specific sites in the 145-bp element, and are implicated in fine-tuning activation of *Myf5* in the forelimb. Msx1, when bound between Pax and Six sites, prevents the binding of these key activators, thus inhibiting transcription of *Myf5* and consequent premature myogenic differentiation. Meox2 is required for *Myf5* activation at the onset of myogenesis via direct binding to other homeodomain sites in this sequence. Thus, these homeodomain factors, acting in addition to Pax3 and Six1/4, fine-tune the entry of progenitor cells into myogenesis at early stages of forelimb development.

## Abstract (precis)

**Summary:** Homeodomain factors Msx1 and Meox2, acting in addition to Pax3 and Six1/4, fine-tune the entry of progenitor cells into myogenesis at early stages of forelimb development via modulation of limb enhancer gene *Myf5*.

## INTRODUCTION

Skeletal muscles in the trunk and limbs derive from myogenic progenitor cells present in the somites of the vertebrate embryo and their formation depends on myogenic regulatory factors controlling muscle cell determination and differentiation (see Tajbakhsh and Buckingham, 2000). *Myf5* is expressed at the onset of myogenesis in the mouse embryo (Ott et al., 1991) when, together with *Mrf4*, it determines myogenic cell fate (Braun et al., 1992; Kassar-Duchossoy et al., 2004). Thereafter, *MyoD* is expressed and can direct cells into the myogenic programme when Myf5 and Mrf4 are absent (Braun et al., 1992). The absence of these three myogenic determination factors leads to the absence of skeletal muscles (Kassar-Duchossoy et al., 2004; Rudnicki et al., 1993). Skeletal muscle in the limbs is formed by muscle progenitor cells that delaminate from the hypaxial dermomyotome of the somites and migrate into the limb field. These cells express the paired/homeodomain transcription factor Pax3 and in its absence they fail to migrate and subsequently undergo apoptosis (see Buckingham and Relaix, 2007). Migration in response to the ligand HGF depends on the c-met receptor (Bladt et al., 1995), and on CXCR4, the receptor for the ligand SDF, which like HGF, is expressed by mesenchymal cells in the limb bud (Vasyutina et al., 2005). The *c-met* gene is a target for Pax3 (Epstein et al., 1996) and *CXCR4* is genetically downstream of *Lbx1* which is also expressed in Pax3-positive migratory cells. In the absence of Lbx1, the ventral muscle mass fails to form and these myogenic progenitors remain in the vicinity of the somite where they can adopt other cell fates (see Buckingham, 2001; Schäfer and Braun, 1999). Six homeodomain factors, like Pax3, are important upstream regulators of myogenesis (see Buckingham and Relaix, 2007) and Six1/4 are also expressed in myogenic progenitors that migrate to the limbs. In the absence of Six1/4, limb muscles do not form correctly and *Pax3* expression in the hypaxial somite is compromised (Grifone et al., 2005).

Transcriptional regulation of the myogenic determination genes has been extensively studied. *Myf5* and *Mrf4* are closely linked on mouse chromosome 10 and their transcriptional regulatory elements extend over a region of at least 120 kb, 5′ to and within the *Mrf4-Myf5* locus. A number of enhancers have been characterised which direct different aspects of the complex spatiotemporal regulation of *Myf5* in the embryo (see Buckingham and Vincent, 2009; Carvajal and Rigby, 2010; Daubas and Buckingham, 2013; Moncaut et al., 2012; Ribas et al., 2011).

A regulatory region required for *Myf5* transcription in the limb and in the more mature hypaxial somite is located at −48/−58 kb 5′ of *Myf5* (Hadchouel et al., 2000). Within this region, complete expression in the developing limbs requires the concerted activity of at least three sub-regions, with the main limb enhancer located at −57/−58 kb (Hadchouel et al., 2000, 2003). Within this enhancer a 145-bp core sequence contains an essential Pax3 paired domain binding site (Bajard et al., 2006) and an adjacent Six1/4 binding site, required for complete activity (Giordani et al., 2007). Mutation in a homeodomain X-vent-type site, between the Pax and Six sites, negatively affects enhancer activity (Buchberger et al., 2007). Pax3 and Six1/4 are expressed in myogenic progenitor cells that migrate into the limb buds prior to *Myf5* activation. Within the developing limb, a proportion of these cells will proliferate, with subsequent expression of *Pax7*, and do not immediately enter the myogenic programme (see Buckingham and Relaix, 2007). Once myogenic progenitor cells have populated the prospective limb muscle masses, Sonic Hedgehog (Shh) drives myogenesis specifically within the ventral muscle mass, to enhance *Myf5* transcription through essential Gli-binding sites located immediately 3′ of the core 145-bp element in the −57/−58 kb enhancer (Anderson et al., 2012). However during the delamination/migration of the myogenic progenitor cells, it is not clear what other regulatory factors interact with the 145-bp sequence to prevent premature activation of *Myf5*. When this sequence is present in multiple copies in a reporter transgene, premature activation is observed, suggesting saturation of potential repressor mechanisms (Bajard et al., 2006). Two other homeodomain factors, Meox2 and Msx1, are also expressed in migratory limb myogenic progenitor cells. In *Meox2* mutants, *Myf5* activation in the limb buds is delayed and there are later muscle defects, attributed to secondary effects of Meox2 in connective tissue (Mankoo et al., 1999). Meox2 binds *in vitro* to the Xvent2 sequence, but when a BAC transgene encompassing the entire *Mrf4/Myf5* locus and regulatory regions was placed in a *Meox2* mutant background no effects on *Myf5-nLacZ* expression were detected (Buchberger et al., 2007). *Msx1* is expressed in mesenchymal cells throughout the early forelimb bud, and also in the myogenic progenitor cells that migrate from the mouse somite to the forelimbs (Houzelstein et al., 1999). In contrast, *Msx2* has a distinct expression pattern but its expression in myogenic cells has not been reported. Msx1 is known to inhibit myogenic differentiation (Song et al., 1992) and *MyoD* activation (Woloshin et al., 1995) when over-expressed in cultured muscle cells and has been shown to recruit the repressive polycomb complex (Wang et al., 2011). ChIP Seq experiments in an *ex vivo* over-expression context, show Msx1 binding to the *MyoD* core enhancer and to the −57/−58 kb *Myf5* regulatory region. Over-expression of Msx1 in the chick limb bud prevented *MyoD* activation and resulted in reduced skeletal muscle formation, with a proposed mechanism through direct repression by Msx1 binding to the *MyoD* core enhancer and also by Msx1/Pax3 complex formation that inhibits Pax3 binding to its targets (Bendall et al., 1999). We therefore decided to investigate more closely the potential role of Msx1 and Meox2 homeodomain factors in modulating the activity of the 145-bp core element of the *Myf5* limb enhancer in myogenic progenitor cells that migrate to the forelimb, at the onset of myogenesis in the mouse embryo.

## RESULTS

### Meox2 and Msx homeoproteins bind *in vitro* to the *145-bp Myf5* enhancer

**Fig. 1.**
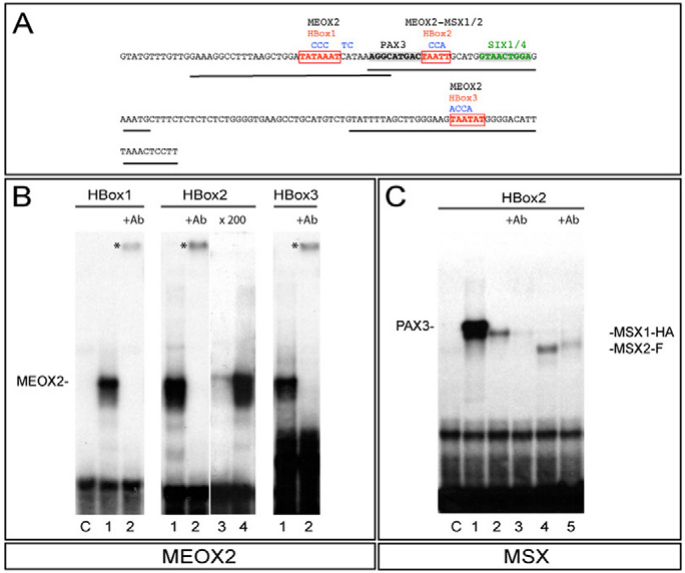
**EMSA experiments showing that the 145-bp *Myf5* element contains HBox sites to which Meox2 and Msx1/2 proteins bind *in vitro*.** (A) The DNA sequence of the 145-bp *Myf5* enhancer. HBox1-3 binding sites are in red. Blue letters above the sequence show nucleotide changes in mutated oligos used in EMSA. HBox1-3 DNA probes used in gel shifts are indicated by solid lines under the sequence. Pax3 (black) and Six1/4 (green) binding sites (Bajard et al., 2006; Giordani et al., 2007) are indicated in bold. (B) EMSA experiments were performed using 3 different DNA probes containing putative HBox1, 2 or 3 binding sites. *In vitro* synthesized Meox2 protein was added to the probes (lanes 1-2) and polyclonal anti-Meox2 antibodies were subsequently added (lanes 2). Each probe binds Meox2 protein and the complex is supershifted when antibodies are added (asterisks). Competition with unlabelled probe is shown for HBox2 (lanes 3, 4). A 200 molar excess of unlabelled probe competes the binding (lane 3), whereas unlabelled probe with a mutated binding site does not compete (lane 4). A control with crude reticulocyte lysate is shown in lane C. (C) Example of an EMSA experiment showing that Msx1 and 2 proteins can bind to the HBox2 probe. Binding occurs when Msx1-Cterm-HA (Msx1-HA) or Msx2-Cterm-Flag (Msx2-F) proteins are present (lanes 2 and 4) and specificity is demonstrated by the reduction of the shifted bands when antibodies, anti-HA (lane 3) or anti-Flag (lane 5), are added. Controls are shown with crude reticulocyte lysate (lane C) or with Pax3 protein as a positive control (lane 1).

We first examined the 145-bp *Myf5* regulatory element for homeodomain consensus binding (HBox) sequences (Noyes et al., 2008), in addition to the binding sites for Pax3 (Bajard et al., 2006) and Six1/4 (Giordani et al., 2007) which are known to be functionally important. Three binding sites, HBox1, 2 and 3 were identified (Fig. 1A), where HBox2 is part of the previously described Xvent2 site (Buchberger et al., 2007) and HBox1 and 3 are located 5′ and 3′ respectively of the central Pax3-HBox2-Six1/4 domain. Electrophoretic mobility shift assays **(**EMSA) show that Meox2 protein interacts with all three HBox sequences (Fig. 1B). Specificity of interaction is shown by the presence of supershifted bands with anti-Meox2 antibodies (Fig. 1B, lanes 2) and by competition with an excess of unlabelled probe, but not with a probe in which the HBox sequence is mutated (see Fig. 1B, lanes 3, 4 for HBox2 example). Meox2 binding is abolished when the HBox sites are mutated (results not shown). Similar experiments with Msx1 and Msx2 proteins show that they bind HBox2 (Fig. 1C, lanes 2 and 4), but not HBox1 and 3 (results not shown). In the absence of reliable Msx antibodies, recombinant proteins with haemaglutinin (C-terminal-HA) or Flag (C-terminal-Flag) tags for Msx1 or Msx2 respectively were used. Antibodies against HA or Flag substantially reduced binding (Fig. 1C, lanes 3 and 5). In this case a supershift is not seen, presumably because the protein/DNA complex is disrupted. In conclusion, Meox2 protein can bind *in vitro* to HBox1, 2 and 3, whereas Msx1/2 binds only to HBox2 of the 145-bp *Myf5* regulatory sequence.

### Meox2 or Msx1 do not bind *in vitro* simultaneously with Pax3 or Six proteins to the HBox2 region

**Fig. 2.**
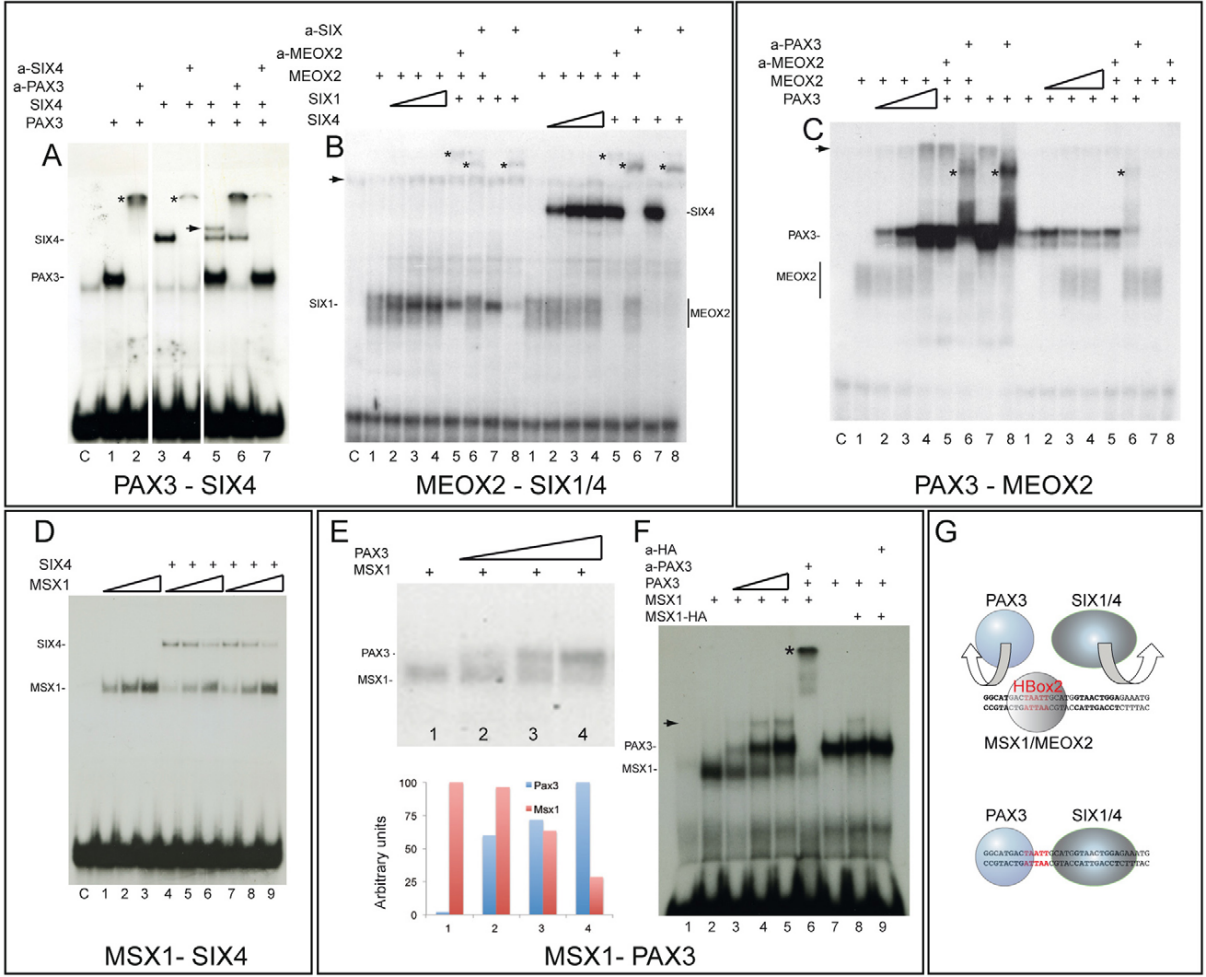
**EMSA experiments with combinations of proteins to test co-binding or competition on the HBox2 probe.** The labelled HBox2 probe, which contains Pax3 and Six binding sites was used in limiting molar amounts, compared to the proteins added. (A) Pax3 and Six4 proteins can bind together to the same DNA sequence. Pax3 binding (lane 1) is supershifted with anti-Pax3 antibodies (lane 2, asterisk). Six4 binding (lane 3) is supershifted when anti-Six4 antibodies are present (lane 4, asterisk). When both proteins are present, an additional slower migrating band is detected (lane 5, arrow) and the intensity of the band corresponding to Six4 binding alone is reduced, compared to lane 3. When anti-Pax3 (lane 6) or anti-Six4 (lane7) antibodies are added, this band, which is therefore due to co-binding of Pax3 and Six4 on the same oligo, is disrupted and the bands due to Pax3 or Six4 binding alone are supershifted. Similar results were obtained with Pax3 and Six1, with disruption of the Pax3/Six1 complex with anti-Pax3 antibodies (results not shown). Lane C is the control with crude lysate. (B) Meox2 and Six1/4 co-binding is not detectable. EMSA was performed with constant amounts of Meox2 and increasing amounts of either Six1 (left side of panel) or Six4 (right side of panel). Presence of anti-Meox2 (lanes 5) or anti-Six1 (left, lanes 6,8) or Six4 (right, lanes 6,8) antibodies are indicated above the panel. No additional slower migrating band, suggesting co-binding of Meox2 and Six proteins, is detected. A weak band (indicated by an arrow) was detected in most samples, including the control with crude lysate (lane C). Asterisks indicate the position of bands supershifted by antibodies. (C) Meox2 and Pax3 co-binding is not detectable. In the presence of constant amounts of Meox2 (left side of panel), no Pax3 (lane 1) or increasing amounts of Pax3 were added (lanes 2-4). No supplementary band is detected when both proteins are present. Controls are shown with Pax3 alone (lane 7) or with Pax3 and anti-Pax3 antibody (lane 8) or lysate alone (C). In the presence of constant amounts of Pax3 (right side of panel), no Meox2 (lane 1) or increasing amounts of Meox2 were added (lanes 2-4). No additional slower migrating band is detected when both proteins are present in the binding reaction. A weak band (arrow) was detected in most samples, including those without Meox2 and appears to represent a Pax3 complex with this lysate. Addition of anti-Meox2 (lanes 5, 8) or anti-Pax3 (lane 6) antibodies disrupt the bandshifts and, in the case of Pax3, generate a supershift (asterisk). Controls are shown with Meox2 alone (lane 7) or with Meox2 and anti-Meox2 antibody (lane 8). (D) Msx1 and Six4 co-binding is not detectable. Increasing amounts of Msx1 alone (lanes 1-3) or with constant amounts of Six4 added at the same time (lanes 4-6) do not produce an additional slower migrating band when both proteins are present. Six4 binding is reduced when higher amounts of Msx1 are added (lanes 4-6). This displacement occurs even if Six4 protein is added before Msx1 (lanes 7-9). (E) Msx1 binding is displaced by increasing amounts of Pax3. When constant amounts of Msx1 without (lane 1) or with 2.5, 5, or 10 fold amounts of Pax3 (lanes 1-4) are added, reduction of Msx1 binding is seen as Pax3 levels increase, under gel conditions which facilitate distinction of Pax3 and Msx1 binding with lower levels of Pax3. This is confirmed by the histogram showing a scan of this autoradiograph with ImageJ 1.47v. (F) Msx1 and Pax3 can bind together when the spacing between the sites is increased. One copy of a mutated HBox2* site, which does not bind Msx1 (see Fig. 4), was intercalated on either side of the bona fide HBox2 site, thus increasing the distance between Pax3 and Six binding sites. In the presence of constant amounts of Msx1 (lanes 2-6) and increasing amounts of Pax3 (lanes 3-5), a supplementary slower migrating complex appears (arrow). When anti-Pax3 antibody is added, this band disappears and Pax3 complexes are supershifted (asterisk, lane 6). This band does not appear if Pax3 is added alone (lane 7) but is detected when Pax3 and Msx1-HA proteins are added together or sequentially (lane 8) and disappears if anti-HA antibodies are added (lane 9). Note that bands corresponding to Pax3 or Msx1-HA complexes alone migrate at almost the same positions as shown in Fig. 1 (G) The drawing resumes the competition between homeoproteins such as Msx1 and Meox2 and Pax3/Six proteins for *in vitro* binding to the oligonucleotide probe in the EMSA experiments presented. The binding of a homeoprotein at the HBox2 site (red) prevents the binding of Pax3 or Six1/4.

The close proximity of Pax3 and Six1/4 binding sites to the HBox2 site, which binds Meox2 and Msx proteins, raises the question of whether these proteins can bind simultaneously. We used limiting molar amounts of labelled DNA probe to test this in gel shift assays. Binding of Pax3 and Six4 together is demonstrated by the appearance of a supplementary slower migrating band (Fig. 2A, lane 5) which is disrupted when either anti-Pax3 or anti-Six4 antibodies are added (Fig. 2A, lanes 6, 7). A similar result was obtained with Pax3 and Six1 proteins (not shown). This indicates that Pax3 and Six proteins can bind together on the same Pax3/HBox2/Six DNA sequence. In contrast, when constant amounts of Meox2 are added, with increasing amounts of either Six1 or Six4, we found no evidence for co-binding on HBox2 and Six binding sites since no supplementary slower migrating band was detected (Fig. 2B). When constant amounts of Meox2 are added with increasing amounts of Pax3, or constant amounts of Pax3 with increasing amounts of Meox2, no supplementary band was detectable and we conclude that Pax3 and Meox2 cannot bind simultaneously on the same DNA sequence (Fig. 2C). Similar experiments with Msx1 and Six4 proteins (Fig. 2D) also indicated that Msx1 cannot bind together with Six4. In these experiments, competition for DNA binding was observed. When increasing amounts of Msx1 are added, Six4 binding is disrupted (Fig. 2D, lanes 4-6). This also occurs if Msx1 is added subsequently, after Six4 binding to the oligo (lanes 7-9). Competition is also observed between Pax3 and Msx1 and is illustrated in Fig. 2E, where increasing amounts of Pax3, relative to a constant amount of Msx1, led to a diminution of Msx1 binding. Again no additional slower migrating band was observed, even on long exposures of the autoradiogram (result not shown). In order to test whether steric hindrance is the cause of this competition, we engineered an elongated version of the probe where a mutated HBox2* sequence, which does not bind Msx but does not interfere with Pax3 binding (see Fig. 4), is introduced on both sides of the bona fide HBox2 site, increasing the distance between the Pax3 and Six1/4 sites. In this case, we can detect an additional slower migrating band, indicating that Pax3 and Msx1 are bound to the same elongated DNA sequence (Fig. 2F). We therefore propose that homeodomain protein binding to the HBox2 site interferes with binding of Pax3, which is essential for the function of the 145-bp *Myf5* regulatory sequence (Bajard et al., 2006). Interference with Six binding will also have a negative impact on the activity of the limb enhancer (Giordani et al., 2007). This may be important in preventing premature activity of the 145-bp sequence in migrating myogenic progenitors which contain Pax3 and Six factors, but in which *Myf5* is not yet transcribed (Fig. 2G).

### Meox2 and Msx1 bind the 145-bp *Myf5* sequence *in vivo*

**Fig. 3.**
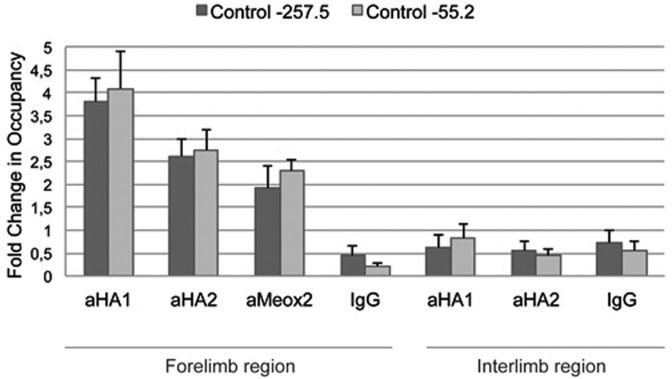
**Msx1 and Meox2 proteins interact *in vivo* with the 145-bp *Myf5* regulatory sequence.** ChIP experiments were carried out with two different anti-HA antibodies (aHA1-2), to immunoprecipitate chromatin prepared from the thoracic region of the trunk including forelimb buds (forelimb region), of E10-E10.5 *Msx1^Tag/Tag^* embryos, or with anti-Meox2 antibodies to immunoprecipitate chromatin prepared from the equivalent region of wild-type embryos at E10.5. Histograms represent the fold change in occupancy of the 145-bp *Myf5* element, versus two different negative control regions located respectively at −257.5 kb (black) and −55.2 kb (grey) upstream of the *Myf5* gene transcription start site (Bajard et al., 2006; Giordani et al., 2007). A control using IgGs from non-immune serum is shown for experiments with wild-type chromatin. Results on the right part of the figure were obtained with ChIP on extracts from interlimb regions, excluding limb buds, of *Msx1^Tag/Tag^* embryos at E10.5, using anti-HA antibodies or control non immune IgGs. Biological replicates of these ChIP experiments were carried out with three different preparations of chromatin. Error bars indicate s.e.m.

We next investigated Msx1 and Meox2 binding *in vivo* to the 145-bp *Myf5* regulatory sequence by chromatin immunoprecipitation (ChIP) experiments, using sonicated chromatin prepared from the thoracic region of the trunk including the forelimbs of embryonic day (E)10-E10.5 mouse embryos. An anti-Meox2 antibody was used on wild-type embryos, and anti-HA antibodies were used to immunoprecipitate HA-tagged Msx1 protein prepared from *Msx1^Tag/Tag^* embryos. The Tag does not interfere with Msx1 function (Duval et al., 2014). ChIP results are presented as a ratio of binding to the 145-bp element at −57.5 kb compared to that obtained with two negative control regions at −275.5 kb and −55.2 kb upstream of the *Myf5* locus, previously used as control for ChIP (Bajard et al., 2006; Giordani et al., 2007). Msx1 binding is not observed with chromatin from interlimb regions excluding limb buds, of E10.5 embryos (Fig. 3). These experiments therefore show *in vivo* binding of Msx1 and Meox2 to the 145-bp sequence at forelimb level.

### Functional importance of the HBox2 site for the correct timing of *Myf5* activation in forelimb buds

**Fig. 4.**
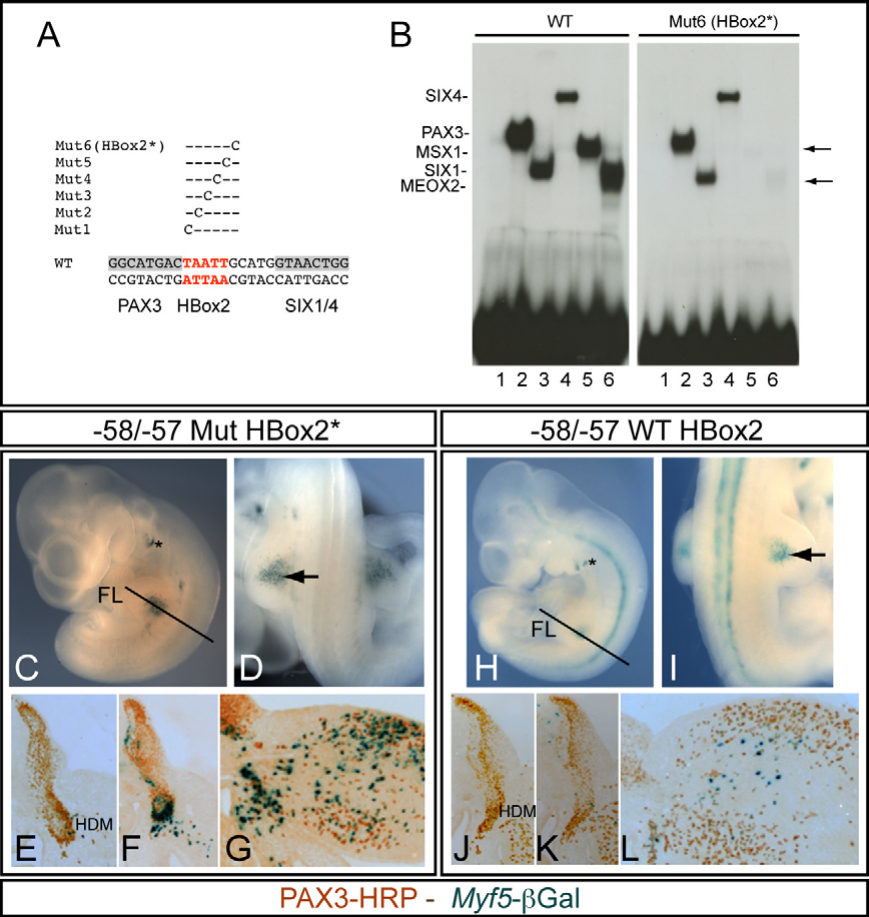
**Mutation of HBox2, without affecting Pax3 and Six1/4 binding, results in premature activation of the −58/−57 kb *Myf5* limb enhancer.** (A). Single mutation scanning in the HBox2 binding site (red) region by successive replacements of nucleotides by a cytosine (C) (Mut1 to 6). Pax3 and Six1/4 binding sites are shown as shadowed boxes. (B) EMSA experiments were performed with the wild-type (WT) sequence or the mutated probe corresponding to Mut6 in order to test the binding of Pax3, Six1/4, Msx1 and Meox2 *in vitro* synthesized proteins. Compared with the wild-type sequence (WT), mutation 6 (Mut6-HBox2*) does not affect the binding of Pax3 (lanes 2), Six 1 (lanes 3) and Six4 (lanes 4), but compromises the binding of Msx1 (lanes 5) and Meox2 (lanes 6) (arrows). Controls with crude lysate are shown in lanes 1. (C-L) Examples of X-gal stained transient transgenic embryos at E10.5 (35/36 somites), obtained with a *−58/−57baMyf5nLacZ* transgene in which the 145-bp sequence within the −58/−57 region contains a mutated HBox2* sequence (C-G) or a wild-type HBox2 (H-L). Whole mount X-Gal stained embryos are shown in C and H, with close-ups of the forelimb region in D and I, where the arrow points to X-gal staining. FL, forelimb bud, asterisk shows branchial arch expression – this and variable neural tube expression are due to sequences in the *Myf5* proximal promoter region used (*baMyf5*). (E-G,J-L) Serial cryostat sections at the forelimb level where the plane of section is shown by a line in C for E,F, and in H for J,K. Sections including the hypaxial somite and proximal forelimb are shown in G and L. These X-Gal stained sections (*Myf5*-β-Gal) were treated with anti-Pax3 antibodies, revealed by horse radish peroxidase (PAX3-HRP) to label Pax3-positive myogenic progenitors (HDM, hypaxial dermomyotome).

*Pax3*, *Six1/*4, *Meox2* and *Msx1* are all expressed in myogenic progenitor cells of the forelimb (Houzelstein et al., 1999; Mankoo et al., 1999; Relaix and Buckingham, 1999) but *Myf5* is not activated until these cells have migrated from the hypaxial somite into the forelimb bud, at the 35 somite stage (E10.5) (Tajbakhsh and Buckingham, 1994). Since both Meox2 and Msx1 proteins bind to HBox2, we investigated the importance of this binding site in regulating the activation of *Myf5*. We screened to find a mutation in or close to HBox2 that prevents Msx1 and Meox2 binding without disrupting the essential binding of Pax3 and Six1/4. Single nucleotide mutations were introduced (Fig. 4A) and the resulting DNA sequences tested for Pax3, Six1/4, Msx1 and Meox2 binding by EMSA. Mutations 2 and 3 abolished both Pax3 and Msx1 binding. Mutation 1 perturbed Msx1 binding and gave additional bands with the reticulocyte lysate. Mutations 4 and 5 both abolished Msx1 binding. In these mutations, Pax3 and/or Six1 binding was also affected (results not shown). This effect on Pax3 and Six1/4 binding probably explains the loss of *Myf5* expression after mutating the Xvent2 site, reported by Buchberger et al. (2007). However we found that mutation 6 (named Mut6-HBox2*) does not perturb Pax3 or Six binding whereas the interaction with both Msx1 (lane 5) and Meox2 (lane 6) was severely compromised (Fig. 4B). The effect of this HBox2* mutation was then tested *in vivo* by transient transgenesis. For these experiments the −58/−57 kb region containing the 145-bp sequence, which gives robust expression, was placed in front of the *Myf5* proximal promoter region and the *nLacZ* reporter. Embryos were collected at E10.5. The HBox2* mutation resulted in premature activation of the transgene, with excessive β-galactosidase labelling in the forelimb bud (Fig. 4C,D) compared to that obtained with the transgene containing a wild-type Hbox2 sequence (Fig. 4H,I). Labelling in the branchial arches and variable labelling in the neural tube is due to sequences present in the proximal promoter region (Carvajal et al., 2001) and was observed with both constructs (Table S2). On serial sections, X-gal staining shows labelled cells within and adjacent to the hypaxial dermomyotome (Fig. 4E,F), whereas such cells are very rare in controls (Fig. 4J,K). A magnified view of a section shows an accumulation of β-galactosidase positive cells adjacent to the somite in the proximal forelimb bud (Fig. 4G), whereas normally only a few dispersed β-galactosidase positive cells are present within the limb (Fig. 4L). Furthermore, with the HBox2* mutated transgene, many more β-galactosidase-positive cells are present within the limb. This indicates that premature activation of the transgene also extends to progenitors within the forelimb, where only a proportion of Pax3-positive myogenic progenitors normally activate *Myf5* and enter myogenesis, while the rest provides a reserve cell population for future muscle growth (Buckingham and Relaix, 2007). We therefore conclude that the HBox2* mutation, which compromises homeobox protein binding, leads to precocious and ectopic activation of the *Myf5* enhancer in myogenic progenitors of the forelimb bud *in vivo*.

### Role of Msx proteins in the early repression of *Myf5* in the hypaxial somite and forelimb bud

**Fig. 5.**
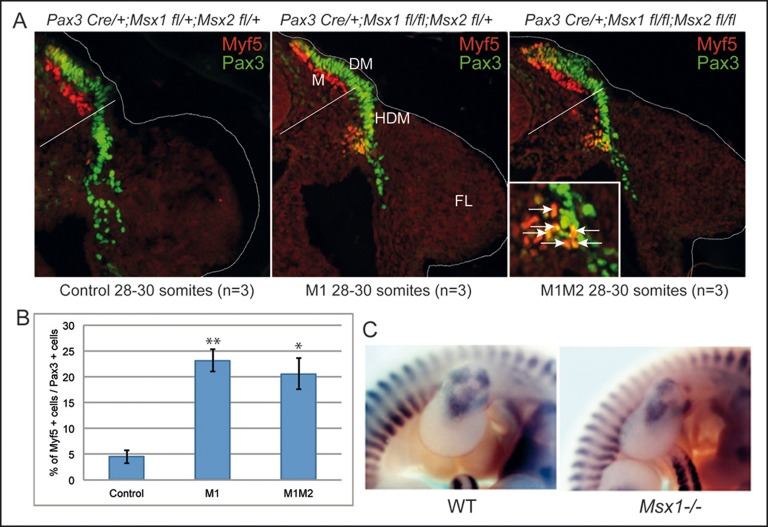
***Msx1* Cre-mediated inactivation in Pax3-positive cells leads to premature expression of *Myf5* in the hypaxial somite.** (A) Immunochemistry on transverse cryosections at forelimb level of *Pax3^Cre/+^;Msx1^fl/+^;Msx2^fl/+^*(control, left), *Pax3^Cre/+^;Msx1^fl/fl^;Msx2^fl/+^* (M1, middle) and *Pax3^Cre/+^;Msx1^fl/fl^;Msx2^fl/fl^* (M1M2, right) embryos at E9.75 (28-30 somites), showing merged images after immunostaining with anti-Pax3 (green) and anti-Myf5 antibodies (red). Contrary to control embryos, double positive Myf5+/Pax3+ cells are found in the region of the hypaxial dermomyotome (HDM), indicated by white arrows in the higher magnification shown for the M1M2 embryo. White lines in merged images represent the upper limit (corresponding to the ventral part of the neighbouring neural tube) below which Myf5+ cells in the Pax3+ population were counted. The contour of the embryos is marked by a white line. FL, forelimb; DM, dermomyotome; M, myotome. (B) Histogram representing the percentage of Myf5+ cells among the Pax3+ cells counted in cryosections equivalent to those shown in (A), with 870-960 Pax3+ cells counted from three embryos for each genotype. Error bars indicate s.e.m. 0.01<*P<0.05; 0.001<**P<0.01; versus control. (C) Comparison of wild-type *Msx1^+/+^* and *Msx1^nLacZ/nLacZ^* (*Msx1^−/−^*) mutant embryos hybridized with an anti-sense *Myf5* riboprobe. Whole mount lateral enlarged views at the forelimb level of embryos at E11.5 are shown.

To test the potential role of the Msx1 protein in the delay of *Myf5* gene activation in myogenic progenitor cells, we examined embryos in which *Msx* conditional alleles had been inactivated specifically in myogenic progenitor cells by the use of a *Pax3^Cre^* allele, at E9.75 (28-30 somites) when they have just begun to delaminate from the hypaxial dermomyotome and enter the forelimb bud. This is the time window when *Msx1* is maximally expressed in the hypaxial dermomyotome at forelimb level (Houzelstein et al., 1999). The efficiency of Cre recombinase to recombine *Msx1* floxed alleles in *Pax3* expressing cells was assessed by *Msx1 in situ* hybridization on cryosections of *Pax3^Cre/+^Msx1^fl/+^* and *Pax3^Cre/+^Msx1^fl/fl^* embryos at E10.5. The control *in situ* signal was too faint in the hypaxial somite, but it was clear that *Msx1* transcripts were absent in *Pax3^Cre/+^Msx1^fl/fl^* embryos, in *Pax3* expressing cells of the dorsal neural tube, a region where *Msx1* is also expressed (Liem et al., 1995), whereas *Msx1* transcripts are present in the non-myogenic distal mesenchyme of the forelimb (Fig. S1). In control embryos at this stage (Fig. 5A, left panel) only rare Myf5-positive (+) cells could be detected in the hypaxial dermomyotome and in adjacent delaminating cells. In contrast, in embryos where both *Msx1* alleles have been recombined, Myf5 expression was observed within the Pax3-expressing population of cells in the hypaxial dermomyotome and in adjacent delaminating cells (Fig. 5A, middle panel). Pax3 expressing cells in the hypaxial region were counted as well as those co-expressing Myf5. 23.2% of Pax3+ cells are also Myf5+ when *Msx1* alleles are inactivated, compared to 4.5% in control embryos. Inactivation of both *Msx1* and *Msx2* alleles gave a similar percentage of Myf5+ cells/Pax3+ cells (20.6%) (Fig. 5A, right panel; Fig. 5B). We also examined sections at forelimb bud level at E10.25 (34-36 somites). Pax3+ cells co-expressing Myf5 are clearly detected in the dorsal-most part of the forelimb bud, and counting of these cells showed a similar tendency between control versus *Msx1* mutant embryos (9.5% of Myf5+ cells/Pax3+ cells, vs 11.8% - results not shown). At later stages, differences in the forelimbs between mutant and wild type were less evident, as shown by *Myf5 in situ* hybridisation for dorsal muscle masses at E11.5 (Fig. 5C). By this stage, as previously shown by Houzelstein et al. (1999), domains of *Myf5* expression in the forelimb are adjacent to those where *Msx1-nLacZ* is expressed, *Myf5* being expressed in future ventral and dorsal muscle masses and *Msx1* in the distal mesenchyme. We conclude that lack of the Msx1 protein *in vivo* leads to premature onset of *Myf5* expression in early Pax3-positive myogenic progenitors as they delaminate from the hypaxial dermomyotome, but does not affect *Myf5* transcription in those cells that continue to migrate into the developing forelimb bud and does not affect later myogenesis.

### *Meox2* is necessary for the early activation of the *145-bp Myf5* regulatory element

**Fig. 6.**
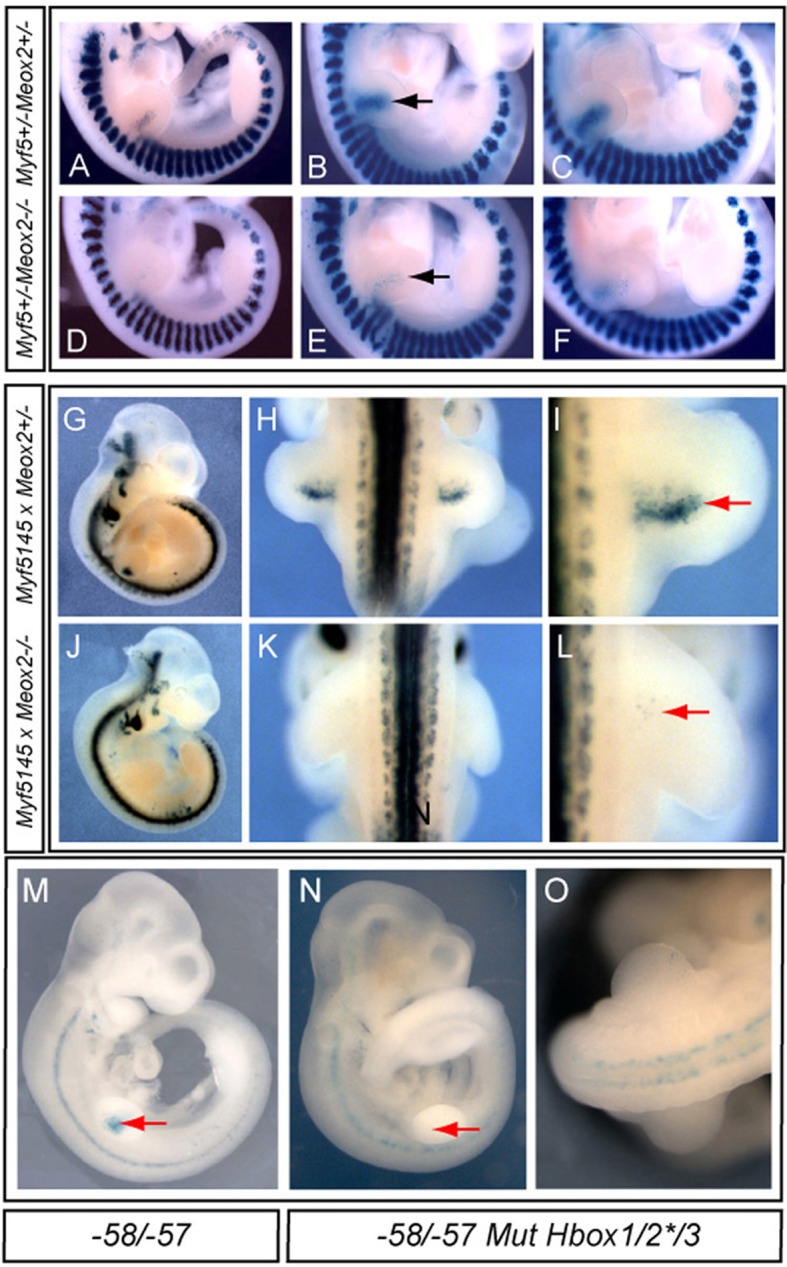
***Meox2* inactivation induces a delay in *Myf5* expression in forelimb buds, which operates via HBox sequences in the 145-bp *Myf5* element.** (A-F) Lateral views showing X-Gal staining at 36 (A,D), 37 (B,E) and 39 (C,F) somite stages of *Meox2^+/−^Myf5^+/nLacZ^* (*Myf5^+/−^*A-C) or *Meox2*^−/−^*Myf5^+/nLacZ^* (*Myf5^+/−^* D-F) embryos. Activation of the *Myf5nLacZ* allele in myogenic progenitor cells in the forelimb bud is delayed in the absence of Meox2 (arrows in B,E). (G-L) X-Gal stained *Meox2^+/−^* (G-I) and *Meox2^−/−^* (J-L) embryos (E10.5) obtained after crossing with a transgenic *145-baMyf5nlacZ* line, with magnifications of the dorsal aspect of the forelimbs shown in H,I,K,L. There is a striking reduction in β-galactosidase positive cells in the mutant at this stage (red arrows). (M-O) X-Gal stained transient transgenic embryos at E10.5, with a *−58/−57baMyf5nlacZ* transgene (−58/−57) (M) or with the same transgene in which HBoxes 1-3 sequences in the 145-bp sequence have been mutated (−58/−57 Mut HBox1/2*/3) (N,O). An enlargement at the forelimb level is shown in O. When all 3 HBox sequences are mutated transgene expression in the forelimb (red arrow) is absent at E10.5. Expression in the branchial arches and neural tube is due to sequences in the proximal promoter region of *Myf5* (*baMyf5*)*,* present in the transgene.

Since the Meox2 protein can bind *in vitro* to the three HBox sites of the 145-bp sequence and because Meox2 is also present in limb myogenic progenitors *in vivo*, we examined the regulation of early *Myf5* expression by this factor. We crossed *Myf5^nLacZ/+^* mice (Tajbakhsh et al., 1996) with the *Meox2^+/−^* mouse line (Mankoo et al., 1999) and examined expression between E10.5 and E12.5, when one or two alleles of *Meox2* are inactivated. There was a clear delay in *nLacZ* expression in forelimb buds of the *Meox2* homozygous mutant versus heterozygous embryos at 36-39 somite stages (Fig. 6A-C compared with D-F). Our observations correlate with the decrease in *Myf5* transcripts reported at E10.5 in forelimb buds of *Meox2^−/−^* mutant embryos (Mankoo et al., 1999). We then crossed a transgenic *145-baMyf5nLacZ* mouse line, with the *Meox2^+/−^* line and observed the same delay in *nLacZ* expression in forelimbs of *Meox2^−/−^* mutants at E10.5 (Fig. 6G-L), indicating that the onset of *Myf5* expression in the forelimbs depends on activation of the 145-bp *Myf5* sequence by Meox2 in Pax3-positive myogenic progenitor cells, which are also clearly present in the mutant (Fig. S2). This delay does not persist at later stages of development, when *145-baMyf5-nLacZ* expression in forelimb buds is observed in *Meox2^−/−^* embryos at E12.5 (Fig. S3), consistent with previous observations (Buchberger et al., 2007). At E12.5, expression of the *145-baMyf5nLacZ* transgene in the hindlimb was delayed in the mutant (Fig. S3). This demonstrates that the mechanism of *Myf5* activation by Meox2 is common to fore- and hindlimbs. In order to determine whether this is a direct effect, we examined transgene function when HBox1, 2 and 3, that bind Meox2, are mutated, using the HBox2* mutation which does not disrupt the binding of Pax3 and Six1/4. These mutations in the 145-bp sequence were tested in the context of the −58/−57 kb *Myf5* enhancer. Compared with the non-mutated *-58/-57baMyf5nLacZ* transgene (Fig. 6M), the *nLacZ* reporter was not expressed in forelimb buds at E10.5 when all 3 HBox sites were mutated (Fig. 6N,O). These results demonstrate the role of Meox2 in the direct activation of *Myf5* through the 145-bp sequence in limb buds at the onset of myogenesis.

## DISCUSSION

The *Myf5* myogenic determination gene is not activated in limb muscle progenitor cells when they delaminate from the somite and migrate to the limb bud, despite expression of Pax3 and Six1/4 which can activate the *Myf5* limb regulatory element. In this context, we show that direct binding of Msx1 and Meox2 controls the precise onset of *Myf5* activation in the forelimb bud *in vivo.* Msx1 prevents precocious activation by competing Pax3 and Six1/4 binding while Meox2 is required to initiate expression once cells have reached the forelimb buds.

We have characterised three homeodomain-binding sites present in this 145-bp sequence, located within an enhancer at −58/−57 kb from the *Myf5* gene. We show that all three HBox sites bind Meox2 *in vitro* and that Meox2 is bound to the 145-bp sequence *in vivo* in preparations from the trunk region, including forelimbs, of E10.5 embryos. At this stage, mutation of all three HBox sites results in a loss of activity of the limb enhancer in *−57/−58baMyf5nLacZ* transgenic embryos. Pax3 and Meox2 proteins are co-expressed in cells that leave the hypaxial dermomyotome and migrate into the limb (Mankoo et al., 1999). These authors also reported delayed activation of *Myf5* transcription in the early forelimb bud in the absence of Meox2. We now confirm this with the β-galactosidase reporter from a *Myf5^nLacZ^* allele and conclude from our transgenic experiments that the HBox sequences in the 145-bp element are implicated in this delay in *–57/–58baMyf5nLacZ* transgenic embryos. This delay is not due to interference with essential Pax3 or Six1/4 binding to adjacent sites, since the HBox2* mutation used permits Pax3 and Six1/4 binding *in vitro* and this mutation alone results in premature activation of the transgene. Pax3 and Meox2 proteins have been shown to interact *in vitro* with each other (Stamataki et al., 2001), however in EMSA experiments we saw no indication of co-binding of Meox2 and Pax3 or Six proteins to the Hbox2 region. In *Meox2* mutants, there is a reduction in *Pax3* transcripts (Mankoo et al., 1999) and a lower level of Pax3 may also impact the −145-bp enhancer, however from our transgenic experiments we conclude that Meox2 directly affects *Myf5* transcription. In the absence of Meox2, the delay in activation of the transgene corresponds to a four somite interval at E10.5. The low level of expression of the *145-Myf5nLacZ* transgene in forelimb buds of *Meox2^−/−^* E10.5 embryos is not due to a lack of myogenic progenitor cells because these are clearly detected at a similar stage using *Pax3* whole mount *in situ* hybridization (Fig. S2). Buchberger et al. (2007), who did not detect a difference in *Meox2* mutants, probably missed the delay we observe at E10.5 by examining E11.5-E13.5 embryos. We found that the delay of *Myf5* activation in *Meox2* mutants, occurring via the 145-bp element, is compensated in forelimbs by E12.5 and indeed no difference in Myosin Heavy Chain expression was observed in *Meox2* mutant limbs (B.S.M., unpublished). At E12.5, the delay is still observed in hindlimbs, which suggests that the mechanism of early *Myf5* activation by Meox2 is similar in fore- and hindlimbs. Since mutation of Hbox1, Hbox2 and Hbox3 sites prevents the correct onset of transgene expression, while the HBox2* mutation alone leads to premature activation of the transgene, we conclude that HBox1 and HBox3 are the sites implicated in activation by Meox2, acting together with Pax3 and Six1/4 on their respective binding sites. This suggests that these factors, and especially Pax3, which is critical for activation, compete favourably over Meox2 binding to HBox2 at the onset of myogenesis, thus avoiding the steric hindrance seen when this site is occupied by Msx1. Our results provide the first demonstration that Meox2 directly activates a skeletal muscle determination gene, fine-tuning the onset of myogenesis in the limb. Subsequently Meox2 is not required in this context and other factors may intervene, such as Mef1 or NFat which also have binding sites in the 145-bp sequence (Buchberger et al., 2007).

Msx1, which is also expressed in myogenic progenitors that migrate to the forelimb (Houzelstein et al., 1999), binds *in vitro* to HBox2, but not to HBoxes 1 and 3. The HBox2 sequence is identical to the consensus site for Msx1 binding to DNA characterized *in vitro* (Catron et al., 1993; Hovde et al., 2001). ChIP experiments confirm that Msx1 binds *in vivo* to the 145-bp sequence, presumably at HBox2, at a stage when myogenic progenitor cells delaminate from the hypaxial dermomyotome and enter the forelimb bud from the somite, prior to *Myf5* activation. *Msx1* is only expressed in the hypaxial dermomyotome at this axial level, as confirmed by ChIP experiments on interlimb somites which show no occupancy by Msx1. Transplantation of early forelimb level somites from *Msx1^nlacZ/+^* embryos into chick embryos at the same axial level showed that β-galactosidase-positive cells subsequently left the somites and entered the limb bud (Houzelstein et al., 1999), leading us to conclude that we are dealing with limb muscle progenitors. We saw no evidence for formation of an inactivating complex between Pax3 and Msx1, in the 145-bp *Myf5* context, as reported previously for the *MyoD* enhancer (Bendall et al., 1999). Instead we show that Msx1 binding to HBox2 competes with binding of Pax3 or Six1/4, respectively to sites 5′ and 3′of HBox2, due to steric hindrance since this is alleviated by increasing the distance between these sites. Thus Msx1 binding to HBox2 prevents binding of essential activators and also probably exerts a direct repressive effect by recruiting Polycomb as shown for *MyoD* (Wang et al., 2011) and/or by recruitment of methyltransferase G9a and repressive H3K9me2 as shown for this *Myf5* regulatory region in C2 muscle cells overexpressing Msx1 (Wang and Abate-Shen, 2012). In transgenic analysis, when HBox2 is mutated, premature activation of *Myf5* in the hypaxial somite region and in myogenic progenitor cells in the forelimb bud is observed, consistent with a role for Msx1 in repressing *Myf5* activation via this site. Expression of the *Msx1^lacZ^* reporter allele in the Pax3-positive myogenic progenitors, is rapidly lost when they reach the proximal region of the limb where myogenic cells accumulate (Houzelstein et al., 1999). Thus we propose down-regulation of *Msx1* releases repression and permits Pax3 and Six1/4 binding. This repression by Msx1 on *Myf5* and also on *MyoD* (Bendall et al., 1999), will effectively prevent the premature onset of myogenesis. When we examine the phenotype of conditional *Msx1^flox/flox^* embryos where the floxed alleles are inactivated by Cre recombinase in Pax3-expressing cells (*Pax3^Cre/+^*), we do not detect a major difference in *Myf5* activation in these cells in the developing forelimb, but we see significantly more Myf5-positive cells in the hypaxial dermomyotome as well as immediately adjacent to this part of the somite at forelimb bud level, at the onset of delamination and migration. Similar results were obtained with *Msx1^flox/flox^; Msx2^flox/flox^* embryos, indicating that Msx1 is primarily responsible and indeed *Msx2* expression has not been reported in somites (Bensoussan et al., 2008). This *Msx1* mutant phenotype is transitory, most evident at the 28-30 somite stage. By E11.5, we did not detect an increase in Myf5-positive cells in the forelimb. In contrast, an increase in *Myf5* transcripts had been reported in the forelimbs of *Msx1* mutant embryos at this stage (Wang et al., 2011), however, since the *in situ* signal was generally higher, it is not clear whether the control and mutant embryos were strictly comparable. Since the effect that we observe is transitory and limb muscle defects have not been reported in the absence of Msx1 (Houzelstein et al., 1997; Satokata and Maas, 1994), it is probable that other factors also intervene to repress premature *Myf5* activation. Up-regulation of the *Myf5* transgene in myogenic progenitors in the forelimb, as well as the hypaxial somite region, when Hbox2 is mutated suggests that another homeodomain binding factor may be involved. Lbx1 is a potential candidate, however we did not observe binding of Lbx1 in EMSA experiments (results not shown). Furthermore, although binding might have exerted steric hindrance, Lbx1 appears to be an activator in the myogenic context (Vasyutina et al., 2005). Other repressive mechanisms may also operate, to prevent premature transcription of *Myf5*. In addition to the 145-bp element within the −58/−57 kb sequence used to test the functional effect of mutating HBox2, a second conserved region contains a Smad binding site, which, when mutated, led to ectopic *Myf5* activation in the vicinity of the somite (Buchberger et al., 2007). Bmp4 in lateral mesoderm adjacent to the hypaxial somite has been shown to prevent premature activation of *MyoD* in the chick embryo (Pourquié et al., 1996) and it is therefore likely that BMP signalling acting through the Smad site also re-inforces repression of *Myf5* transcription during the migration of myogenic progenitor cells to the limb. In these cells, *MyoD* transcription has also been shown to be regulated by transcriptional repression exerted by the bHLH-PAS transcription factor Sim2, acting on the *MyoD* core enhancer (Havis et al., 2012). Repression of *Myf5* was not observed when Sim2 was overexpressed under conditions where *MyoD* was downregulated in chick and Xenopus embryos (Havis et al., 2012). This suggests that the two myogenic determination genes are repressed by a combination of different mechanisms, to prevent premature entry into the myogenic programme.

**Fig. 7.**
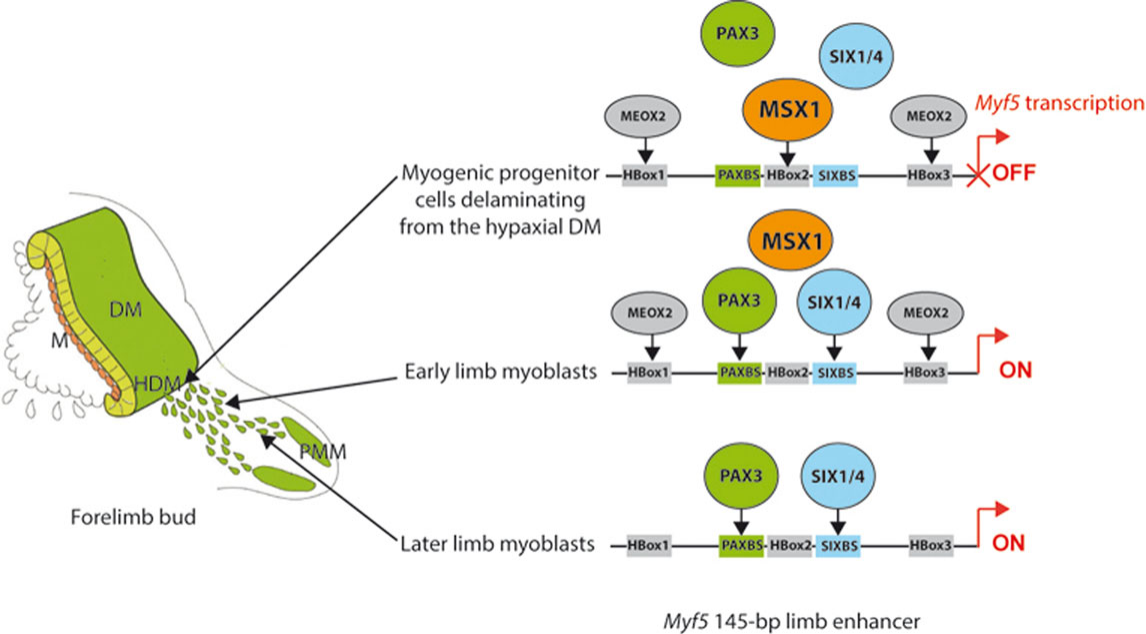
**Model for activation of *Myf5* transcription via the 145-bp core element, during early forelimb bud development.** Binding of Msx1 interferes with binding of Pax3 and Six1/4 to the enhancer, and this prevents premature activation of Myf5 expression in myogenic progenitors in the hypaxial somite. Binding of Pax3 and Six1/4 in conjunction with Meox2 binding to flanking homeobox sites in the enhancer is required for normal activation of Myf5 expression. Maintenance of Myf5 expression in the myoblasts once they have migrated into the limb is dependent on Pax3 and Six1/4 but is independent of Meox2 activity. Abbreviations: DM, dermomyotome; M, myotome; HDM, hypaxial dermomyotome; PMM, pre-muscle masses.

In conclusion, fine-tuning of the onset of *Myf5* activation in myogenic progenitor cells that migrate from the somite to the forelimb depends on the binding of homeodomain factors that inhibit or activate transcription via the 145-bp regulatory sequence at −57.5 kb. We propose that inhibition is normally exerted by binding of Msx1 that interferes with the binding of Pax3 and Six1/4 and may also act as a transcriptional repressor. This prevents premature activation of this myogenic determination gene in Pax3-positive progenitor cells which would compromise the correct localisation of skeletal muscles and also the maintenance of myogenic progenitors required for subsequent muscle growth. In addition to Pax3 and Six1/4 activation of the 145-bp limb element, the initiation of *Myf5* transcription also depends on Meox2 that binds to additional homeodomain sites. A model recapitulating the onset of *Myf5* gene activation during forelimb development is shown in Fig. 7. This example, for a myogenic determination gene, illustrates the importance of transcriptional fine-tuning to ensure the precise spatiotemporal expression of key regulatory factors during development.

## MATERIALS AND METHODS

### Plasmid constructions used for transgenesis

All plasmid constructs used for transgenesis are derived from plasmid *pbaMyf5-nLacZ* (Hadchouel et al., 2000), *p−58/−57* and *p−145-baMyf5nLacZ* (Bajard et al., 2006; Hadchouel et al., 2003) constructs. Mutagenesis of homeodomain binding sites (HBox) was first performed by PCR amplification with the Expand High Fidelity PCR System (Roche), using as a template a plasmid in which the −58/−57 fragment had been subcloned into the pGEMT-Easy vector (Promega) and two primers, forward and reverse, complementary to their 5′ extremities as described in Daubas et al. (2009). Mutations were successively carried out in HBox2, 3 and 1 using primers as described in Table S1. Then a HBox2* mutation was introduced into the −58/−57 fragment, mutated in HBoxes 1, 2 and 3 using a primer HBox2* (see Table S1) and the QuickChange Multi Site-Directed Mutagenesis kit (Stratagene). Mutations were checked by DNA sequencing (GATC Biotech) and the mutated fragments were isolated from pGEMT-Easy plasmids (Promega) and recloned into the *pbaMyf5-nLacZ* vector.

### Mouse lines, transgenesis and embryo analysis

The *Meox2^+/−^* and *Msx1^nLacZ:+^* mouse lines have been described in Mankoo et al. (1999) and Houzelstein et al. (1997). The *Msx1^Tag/Tag^* mouse line was produced by inserting HA Tag in-frame in front of the *Msx1* stop codon followed by an IRES-nls mCherry cassette. After ES cell selection and blastocyst injection, F1 mice born from germline chimaeras were bred with FLPe transgenic mice to remove the neomycin cassette. Heterozygous *Msx1^Tag/+^* and homozygous *Msx1^Tag/Tag^* mice were viable and fertile, indicating that the *Msx1Tag* allele is functional (Duval et al., 2014). Immunodetection of the HA Tag in E10.5 *Msx1^Tag/+^* embryos showed correct expression. Conditional *Msx1* (a kind gift of Dr Robert Maxson, Los Angeles, CA, USA) and *Msx2* floxed alleles are respectively described in Fu et al. (2007) and Bensoussan et al. (2008). The *Pax3^Cre/+^* mouse line is described in Engleka et al. (2005). Transgenic mice were obtained on a C57BL/6JxSJL genetic background. Experiments were performed in accordance with the European Community guidelines (2010/63/UE) and with French national regulations for the care and use of laboratory animals. All transgenesis experiments were carried out by injecting vectorless plasmid inserts and performed by the Centre d'Ingénierie Génétique Murine of the Pasteur Institute. The *−58/−57baMyf5nLacZ* transgenic mouse line was as described previously (Hadchouel et al., 2003). Hetezygous transgenic males were crossed with non-transgenic females (C57BL/6JxSJL F1). Embryos were staged taking E0.5 as the day of the vaginal plug and somites were counted for more precise staging. Most functional studies were carried out by transient transgenesis. Embryos were screened for transgene expression by X-Gal staining as described in Tajbakhsh et al. (1996). The number of transgenic embryos analysed in each experiment is indicated in Table S2.

### Electrophoretic mobility shift assays

A 1044 bp *Sph*I-*Xho*I fragment corresponding to mouse *Meox-2* cDNA was blunted and cloned into the *Eco*RV site of the pcDNA3 plasmid (Invitrogen). The Pax3 expression plasmid was a gift from F. Relaix (Paris Est-Créteil University, Créteil) and consisted of a full length mouse *Pax3* cDNA cloned into a pcDNA3.1 vector (Invitrogen). Six1 and Six4 expression vectors were gifts from P. Maire (Institut Cochin, Paris). Full length *Six1* and *Six4* cDNAs were cloned into the pCR3 CMV T7 expression vector (Invitrogen), as described in Spitz et al. (1998). Plasmid constructions for recombinant Msx1-C-HA and Msx2-C-Flag proteins were designed by N.D. Fragments corresponding to full length coding regions of Msx1-C-HA and Msx2-C-Flag were subcloned into pcDNA3.1+ (Invitrogen) and pCMV-TnT (Promega) vectors, respectively. Expression plasmid DNAs were used to synthesize proteins *in vitro* with the TnT T7 Quick Coupled Transcription/Translation System (Promega). Electrophoretic mobility shift assays (EMSA) were performed as described in Bajard et al. (2006). P^32^-γATP labelled oligos, containing HBox1, 2 or 3 are listed in Table S1, as well as the elongated core enhancer probe, LongPaxHBox2Six (containing spacers between the HBox2 site and Pax3/Six binding sites). Except when specified otherwise, proteins to be tested together were added simultaneously. One microliter of either anti-HA (Roche Applied Science), anti-FLAG M2 (Sigma), rabbit polyclonal anti-Meox2 (B. Mankoo), mouse monoclonal anti-Pax3-C (DSHB) or rabbit anti-Six4 (Sigma) were added to EMSA binding reactions for supershift assays.

### Immunocytochemistry

Immunodetection was performed on X-Gal stained 20 µm cryostat sections, using monoclonal mouse anti-Pax3-C antibodies (DSHB) and the Vector M.O.M (Mouse on Mouse) Peroxidase kit (Vector Laboratories). Sections of control and *Msx* mutants shown in Fig. 5A were 10 µm thick. Monoclonal mouse anti-Pax3-C antibodies (1:250, DSHB), rabbit polyclonal anti-Myf5-C20 (1:250, Santa Cruz), Alexa Fluor 488 Anti-Mouse IgGs and Alexa Fluor 546 Anti-Rabbit IgGs (1:500, Molecular Probes) were employed for immunofluorescence experiments. Nuclear staining was with Hoechst solution.

### *In situ* hybridisation

Whole-mount *in situ* hybridization on mouse embryos was performed as described in Daubas et al. (2000) using digoxigenin-labelled antisense *Myf5* (Ott et al., 1991) or *Pax3* (kindly provided by Dr P. Gruss, Max Planck Institute, Gottingen) riboprobes. Automated *in situ* hybridization on cryostat embryo sections was performed with an InsituPro VSi apparatus (Intavis Bioanalytical Instruments) using a digoxigenin-labelled antisense *Msx1* riboprobe (Lyons et al., 1992).

### ChIP-qPCR

The trunk at forelimb level, including forelimbs, or interlimb regions were dissected from E10-10.5 mouse embryos and the protocol used for chromatin immunoprecipitation (ChIP) and qPCR analysis was as described in Daubas and Buckingham (2013). The following antibodies were used: rat monoclonal anti-HA (high affinity, clone 3F10, Roche – aHA1), rabbit polyclonal anti-HA (ChIP grade, Abcam – aHA2), rabbit polyclonal anti-Meox2 (B.S.M.), rabbit polyclonal anti-histone H3 (tri-methyl K4) (ChIP grade, Abcam) and IgG from rabbit serum (Sigma). qPCR primer sequences are listed in Table S1. All analyses were carried out in 96-well plates using a StepOnePlus PCR machine (Applied Biosystems) and the FastStart Universal SYBR Green Master (Rox) (Roche). qPCR reactions were performed in triplicate with each of 3 different preparations of chromatin prepared either from forelimb or interlimb regions of pooled embryos. 5 µl of DNA solution were used per reaction, corresponding to 1 or 0.1 µl of immunoprecipitated DNA or Input DNA, respectively. Standard curves of all primers were performed to check for efficient amplification (above 90%). Melting curves were also performed to verify production of single DNA species with each primer pair. Relative levels of expression in each assay were obtained through the ΔΔCt method (Livak and Schmittgen, 2001). Fold changes in occupancy, compared to negative control regions, are equal to 2^−ΔΔCt^.
